# Topological indexes and community structure for urban mobility networks: Variations in a business day

**DOI:** 10.1371/journal.pone.0248126

**Published:** 2021-03-10

**Authors:** Jéssica D. Lamosa, Lívia R. Tomás, Marcos G. Quiles, Luciana R. Londe, Leonardo B. L. Santos, Elbert E. N. Macau

**Affiliations:** 1 Instituto Nacional de Pesquisas Espaciais (INPE), São José dos Campos, Brazil; 2 Centro Nacional de Monitoramento e Alertas de Desastres Naturais (Cemaden), São José dos Campos, Brazil; 3 Universidade Federal de São Paulo (UNIFESP), São José dos Campos, Brazil; 4 Humboldt University of Berlin, Berlin, Germany; Monash University, AUSTRALIA

## Abstract

Topological analysis and community detection in mobility complex networks have an essential role in many contexts, from economics to the environmental agenda. However, in many cases, the dynamic component of mobility data is not considered directly. In this paper, we study how topological indexes and community structure changes in a business day. For the analyzes, we use a mobility database with a high temporal resolution. Our case study is the city of São José dos Campos (Brazil)—the city is divided into 55 traffic zones. More than 20 thousand people were asked about their travels the day before the survey (Origin-Destination Survey). We generated a set of graphs, where each vertex represents a traffic zone, and the edges are weighted by the number of trips between them, restricted to a time window. We calculated topological properties, such as degree, clustering coefficient and diameter, and the network’s community structure. The results show spatially concise community structures related to geographical factors such as highways and the persistence of some communities for different timestamps. These analyses may support the definition and adjustment of public policies to improve urban mobility. For instance, the community structure of the network might be useful for defining inter-zone public transportation.

## 1 Introduction

Urban mobility is the whole of commutes of the inhabitants of a city, and the methods and conditions associated with them (modes of transport selected, length of trip, time spent in transport, etc.) [1]. The information on displacements and interactions of people in urban regions support studies on the evolution of cities along time and their main characteristics. The complexity of simultaneous activities happening in cities with thousands of buildings, commerce, industry, and intense displacements between regions highlights the necessity of planning the urban growing [2–4].

Commuting is a significant component of mobility in general. It is a vital target of transport policy and urban planning due to its regular patterns, related issues concerning traffic jams, and links with people’s choices of workplace and residence locations [5–7]. Many studies have shown that urban spatial structure and the spatial relationship between jobs and housing are strongly correlated with commuting patterns. There is also a clear connection between transport energy use and carbon emissions [8, 9]. A comprehensive review of urban mobility models and applications was presented in [10].

There are many ways to investigate the characteristics of cities and mobility. Complex Network, i.e., a structure in which connections (edges) link pairs of elements (nodes), have been applied in several works about urban mobility [11–16]. However, in those previous works, the dynamic component of urban mobility data was considered directly neither for topological indexes nor for community structure.

Vazquez-Prokopec et al. [17] presented a study in dynamic mobility network inferred from GPS data-logger data. Authors analyzed time-series of some topological indexes, such as the largest component’s size, using data from 6 am to 11 pm. From a community detection perspective, Yildirimoglu and Kim [18] compared the spatial structure in three distinct layers (related to buses, passengers, and cars), using accumulated data from 6 am to 10 am. Using anonymous telephone data with the geographical and temporal aggregation, Botta, and del Genio [19] showed that the structure might create challenges for the geographical analysis of communities. They found patterns for the evolution of communities with evidence for circles and social groups and not only individually. The authors suggested pattern trends that may be used to detect critical social events. Information about people’s behavior and their interactions can also be extracted from the community structure of networks.

Exploring communities using spatial and time dynamics is important because: 1) these dynamics may show a spatial structure for displacements, 2) it is possible to verify whether patterns are persistent with time variation, and 3) relations among different nodes and the spatial-time dynamics support the understanding of dynamic processes in the network (i.e., urban planning, communication nets, the spread of epidemics and strategies for events) [20, 21].

In this article, we work on an urban mobility network with spatial and dynamic components. The input data is an Origin-Destination (OD) survey conducted for São José dos Campos municipality, Brazil. This data is a picture of commutes on a business day. Different graphs represent the dynamics and spatial components of the city. We conducted an investigation of topological indexes and community structures in a dynamic and static way to verify how the network’s properties behave throughout the day. We also analyzed the existence and persistence of patterns.

Our results indicate that the proposed approach is worthy and can contribute to developing new mobility rules and policies. Moreover, the mobility network’s community structure might also be suitable for implementing epidemic controls, such as imposing transportation restrictions between zones. Also, the same community structure might be used for defining the rules for inter-zone public transportation.

This paper is organized as follows. Section 2 presents the mobility dataset and the methods taken into account in this research. Results and Discussions for the static and dynamic scenarios are detailed in Section 3. Finally, Section 4 draws some concluding remarks and points out future researches.

## 2 Materials and methods

The adopted methodology can be summarized in three steps: 1) data preprocessing, which consists of generating the OD network using a threshold-based approach; 2) a processing phase that computes the topological properties and the community structure of the network; 3) the creation of a *shapefile* with the topological properties of the graph.

### 2.1 Origin-destination data

The input data comprises the Origin-Destination research (OD Research) database and its products for the municipality of São José dos Campos, Brazil. The population of São José dos Campos is about 721,944 inhabitants, and the municipality occupies an area of 1,100 km², of which nearly 356 km² are urban [22].

**Fig 1:**
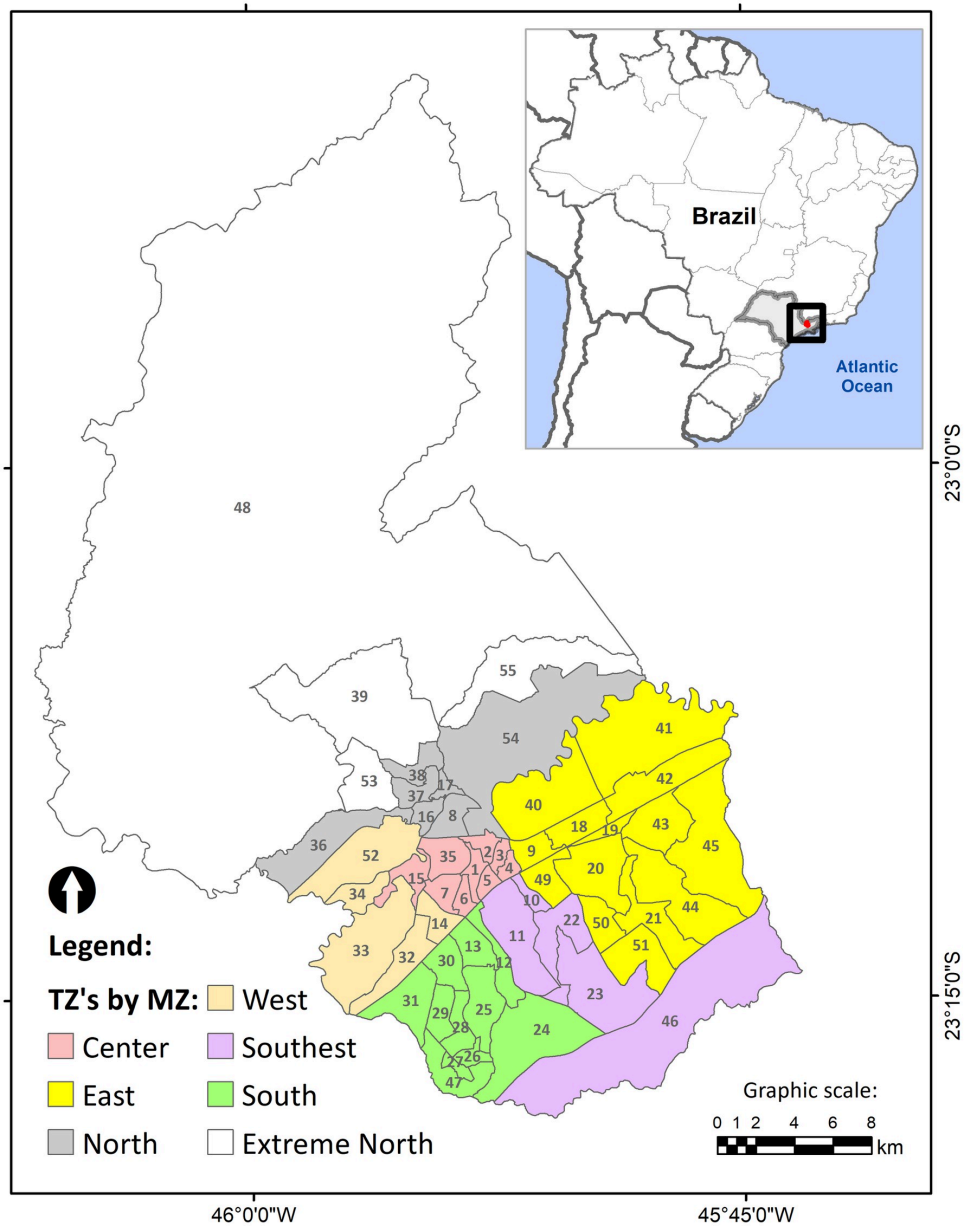
Traffic zones by macrozones.

In 2011, the city hall of São José dos Campos carried an OD research in the municipality’s urban and rural zones. The city was organized into 55 areas named Traffic Zones (TZs) to survey and analyze the collected data. These TZs were obtained by dividing the municipality area into smaller units, according to some criteria that consider existing TZs, census tract districts, the road system, other natural and physical barriers, and homogeneous characteristics of land use and occupation [23]. Another unit used in the research is the macro-zone (MZ), formed by the grouping of TZs. Fig 1 presents the TZs grouped by MZs.

The OD research is a dataset of information on citizen’s displacements. These displacements are related to work, business, leisure, schooling, and routine activities. The information also includes the commuting time and periods of the day in which the travels take place [24].

The official demographic census conducted by the Brazilian Institute of Geography and Statistics (IBGE) was aggregated by TZ to define the universe [22, 25]. The sample size was calculated using the universe by TZ. Households were randomly selected for research, and the residents of these households answered about trips made the day before the survey, representing a business day. The interviews were conducted from Tuesday to Friday. The expanded database sums a total of 1,589,456 trips, with origin and destination in São José dos Campos, made by 627,727 people [23].

### 2.2 Complex networks generation and measurements

**Fig 2:**
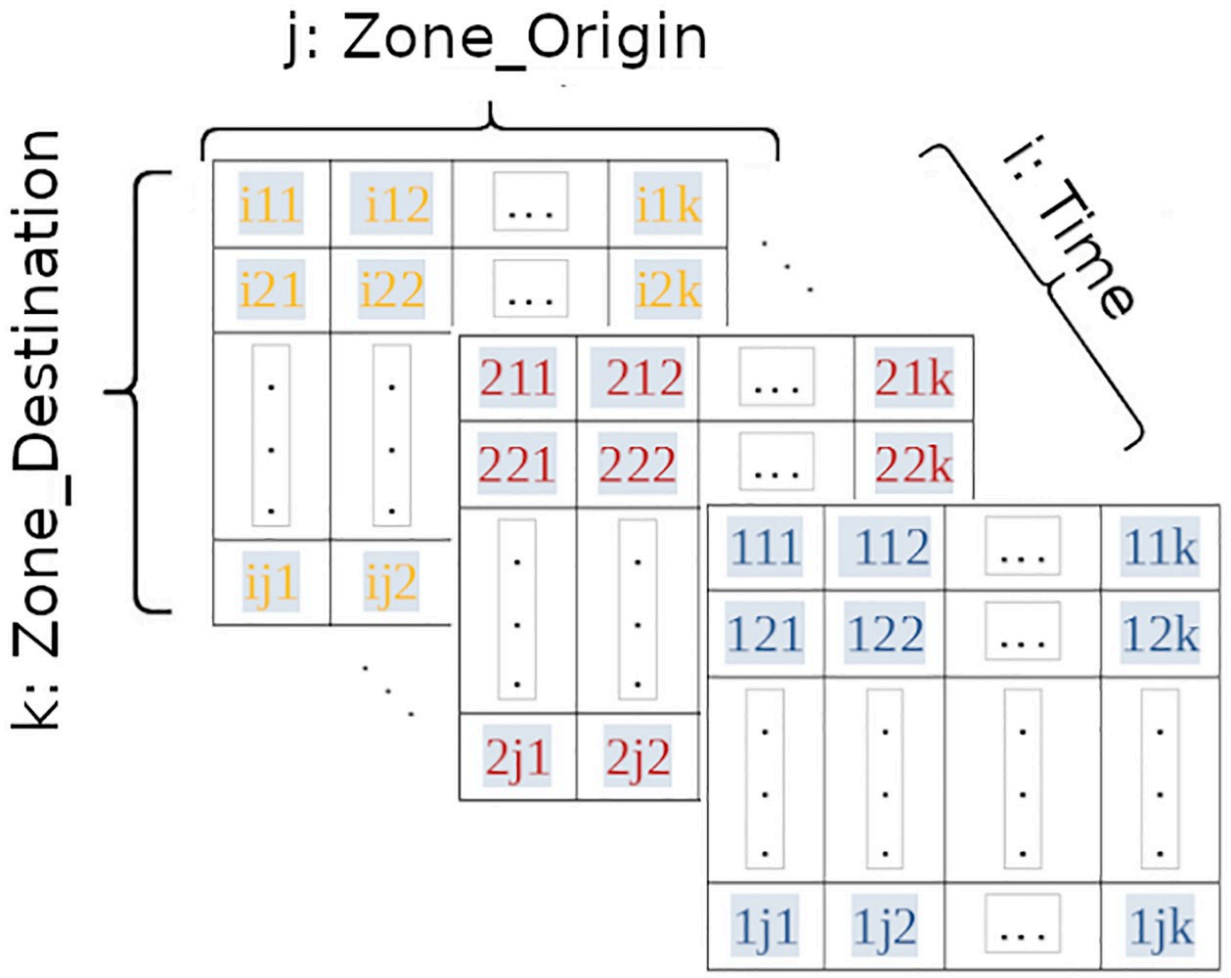
3-dimension matrix.

The commutes between TZs along the day were analyzed using complex networks [26]. Using the database, an OD graph was created, in which each vertex represents a TZ (55 vertices), and the edges are weighted by the number of commutes (connections between TZs). The connection between an origin and a destination forms an OD pair. Static and dynamic networks were considered in this study. They are represented by 3-dimension matrix, as Fig 2 shows.

The *j*-dimension represents the origin with 55 column, *k*-dimension represents a destination, with also 55 lines and *i*-dimension represents the dynamic component. The cell value is set with the number of commutes between zones.

We defined the following rule for creating the network: an edge connects nodes if the number of commutes is greater than the threshold. Two approaches were investigated, threshold (*η*) considering *η* equals one and the other considering a critical threshold, *ηc*. This value is intended to maintain only links associated with a high number of commutes. To establish this critical value (*ηc*), we evaluate networks generated with a range of thresholds and select the one that leads to a single-component network with the largest diameter.

The static network is built considering all the commutes in the 24 hours interval. For *η* equals 1, all connections with at least one commute are considered. On the other hand, with the threshold *ηc*, only connections between nodes with weights, or a number of commutes, greater or equal to the threshold were considered.

A symmetrization approach was also considered to build an undirected network. Thus, the weight between nodes *i* and *j* is defined as the sum of commutes from *i* to *j* and from *j* to *i*. Intra-zone commutes, represented by the main diagonal of the weight matrix, were not considered in this study.

To generate the dynamic networks, we firstly preprocessed the data considering three distinct time-windows: 15, 60, and 180 minutes. After scrutinizing the data, we adopted the 60-minutes time-window as the standard value for generating the dynamic network in this investigation.

A dynamic weighted network was created from the preprocessing, representing hourly ordered sequences, over the fixed set of vertices (TZs). The networks were created with the *igraph* library using the C language. Fundamental measurements were computed from these networks, such as average degree, agglomeration coefficient, and diameter of a graph. These networks were also symmetrized accordingly to the rule defined for static networks.

Table 1 summarizes the topological measurements evaluated in this study. *N* represents the number of nodes in the graph. *M* depicts the number of edges in the graph (number of connections between the TZs). Node degree and clustering are represented by *k* and *c*, respectively. *l* quantifies the shortest path of the graph, and *d* is the diameter of the network.

**Table 1:** Topological properties.

indexes	Meaning
*N*	Number of nodes (Number of TZs)
*M*	Number of edges (Number of connections between TZs)
*k*	Degree of a node (number of connections)
〈*k*〉	Average degree of the network
*c*	Clustering Coefficient
*l*	Average shortest path
*d*	Diameter of the network
*Q*	Modularity

Besides the measurements described before, we also perform a mesoscale analysis of their community structure networks. For this purpose, we applied the *walktrap* algorithm [27], which is a fast community detection algorithm based on random walks. It is worth noting that other community detection algorithms could be used to perform this analysis [28]. The community detection method’s outcome is evaluated with the modularity quality measure *Q* [29], which gives a value between −1/2 and 1. The larger *Q* is, the better is the division of the network in the community. According to [30], values of *Q* ≤ 0.3 indicates a good partition of the network.

To analyze complex networks, the open-source tool GeoCNet (Geographical Complex Networks) was developed. This tool is a Python application with spatially extended that creates (geo)graphs with topological properties, making it possible to generate *shapefile* files for network visualization in a GIS. In this work *QuantumGIS* was used. The tool is available in [31]. Phython3 language was used in data postprocessing, with communication with PostgresSQL database and PostGIS extension.

Each stage of development can be verified in protocols.io [32].

## 3 Results and discussion

This section presents the main results of this work. Specifically, the results and discussion are split into two parts: static networks and dynamic networks. In both cases, we show the results considering the thresholds *η* and *ηc*.

### 3.1 Static network

Considering the static network with *η* equals 1, there are three neighbor nodes with the highest topological degree in this network (*k* = 49): nodes 1 and 2 (both in downtown) and node 8 (in the north region). The region associated with node 8 is one of the most traditional parts of the city, with several services (banks, drugstores, schools) working as an expanded-center area. The highest possible topological degree in this network is 51, once there are 52 reachable nodes (3 nodes belong to protected zones with no allowed commutes). These three zones (1, 2, and 8) are connected to almost all the other city zones (96% of the network); thus, they can be considered the city-center from a topological point of view.

Table 2 summarizes the average and global indexes for the static network with *η* equals 1 in left column. The average topological degree (〈*k*〉) is very high (64% of the highest possible value: 51), the average clustering coefficient is very high as well (80% of the highest possible value: 1), the diameter is very low (merely twice the lowest possible value: 1). The modularity *Q* = 0.209 indicates that the community structure of the network is not prominent, as a value of at east 0.3 is expected [30].

**Table 2:** Topological properties of the static network.

Indexes	*η* = 1	*ηc* = 6,231
〈*k*〉	32.7	0.36
〈*c*〉	0.8	0.0
*D*	2	6
*Q*	0.209	0.410

The right column of Table 2 summarizes the average and global indexes for the static network with *ηc* equals 6,231, representing the threshold established with the rule introduced in Sec. 2.2. In this scenario, the average degree (〈*k*〉 = 0.36) indicates a very sparse network. The clustering coefficient *c* = 0.0 is related to a tree-like structure of the network. The diameter of this network is *d* = 6 and the modularity *Q* = 0.41, which indicates that the community structure is more prominent in contrast to the network generated with threshold *eta* = 1.

**Fig 3:**
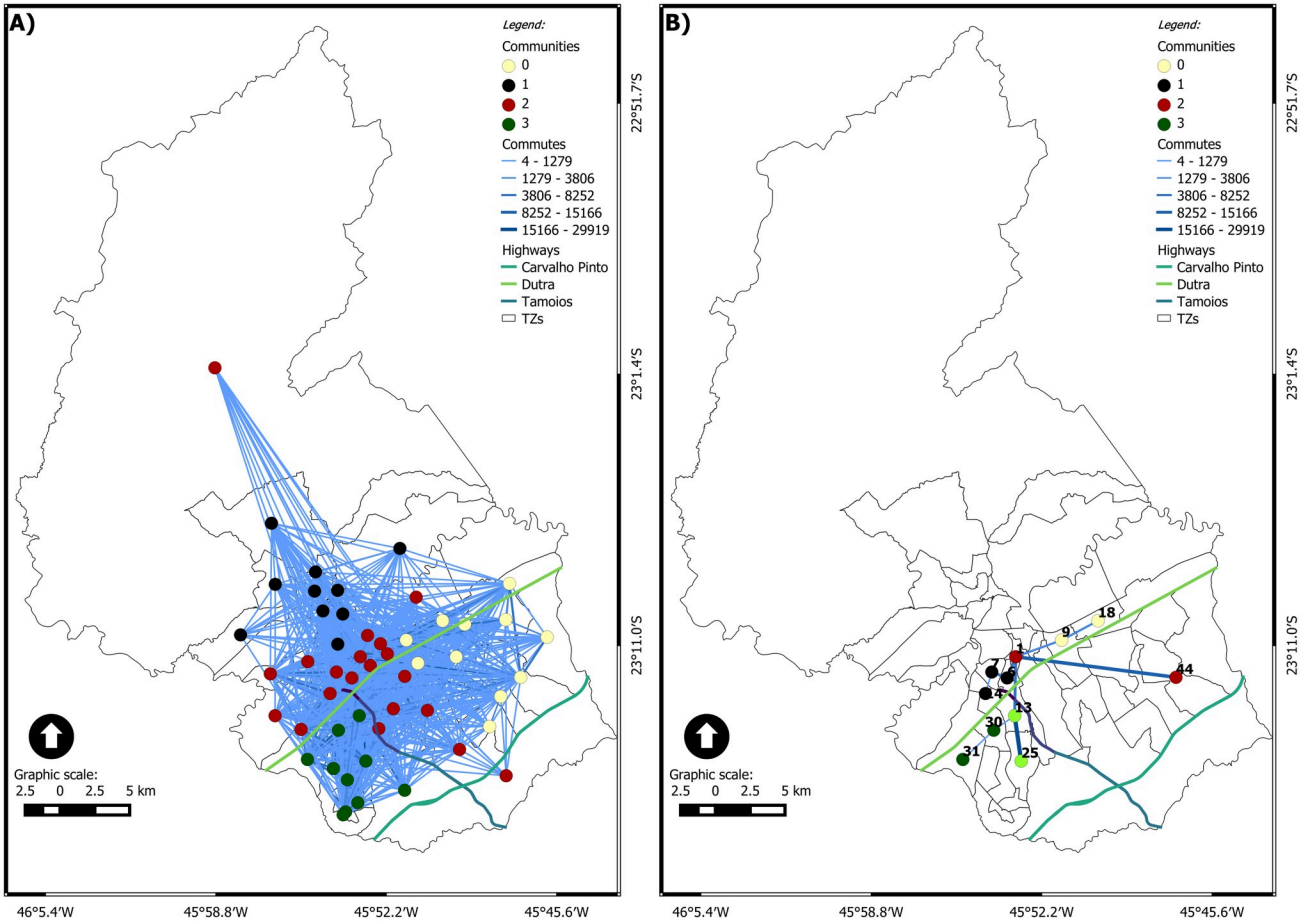
(Geo)graph of the accumulated of the day of the São José dos Campos municipality. (A) *η* = 1 and (B) *ηc* = 6, 231.

Fig 3(A) shows the static network with *η* equal to 1, following the (geo)graphs approach. It is possible to visualize the community structure, with four spatially concise communities, regarding the regions north, south, east, and center. In this case, the modularity *Q* is equal to 0.209. The topological center area’s community brings together the nodes in the geographical center and the nodes in the west, southeast, and the node in the extreme north area. The node in the extreme north area (TZ 48) represents a very interesting case, as it is closer (in a topological point of view) to the center area than to the north area. TZ 48 is mainly composed of a rural area and a small urban area belonging to a district of Sao José dos Campos: São Francisco Xavier. The internal commutes of this TZ are the most expressive: the people that live there do their daily activities within the TZ. Considering the commutes to other TZ, the strongest connection is with TZ 1 (the most central zone). It represents 35% of the total commutes, and 70% of external commutes are for a node in the central region. The main reasons for these commutes are work, return to home, health, and shopping.

Fig 3(B) shows the static network with *ηc* equal to 6,231, following the (geo)graphs approach. In this case, there are just five concise communities, each one with two or three nodes only, and *Q* is equal to 0.41. The node 1, in downtown, is connected to nodes in 3 of the 4 other communities.

For the static network with *ηc* equal to 6,231 (Fig 3B), the size of the largest connected component is reduced from 51 to 11. The node 1 is the most connected one, but *k*_1_ = 4, only. It is interesting to highlight that the connected component presents a tree-like structure. Therefore, on the one hand, the critical connection threshold reveals a significantly reduced set of nodes in the largest connected component. On the other hand, it keeps the core-structure of the network.

### 3.2 Dynamic network

For the dynamic network analysis, as stated before, we consider a one-hour time window, from 0 am to 11 pm: 24 boxes, each one of them generating one network, accumulating all travels starting in the specified time window. There is a time series for topological indexes, such as average topological degree, average clustering coefficient, and network diameter, associated with each connection threshold: *η* = 1 and *η* = *ηc*.

Where ‘commutes’ represents the accumulated commutes, ‘diameter’ represents the diameter of the network, ‘clustering’ represents the average clustering coefficient of the network, and ‘degree’ represents the average degree of the network.

**Fig 4:**
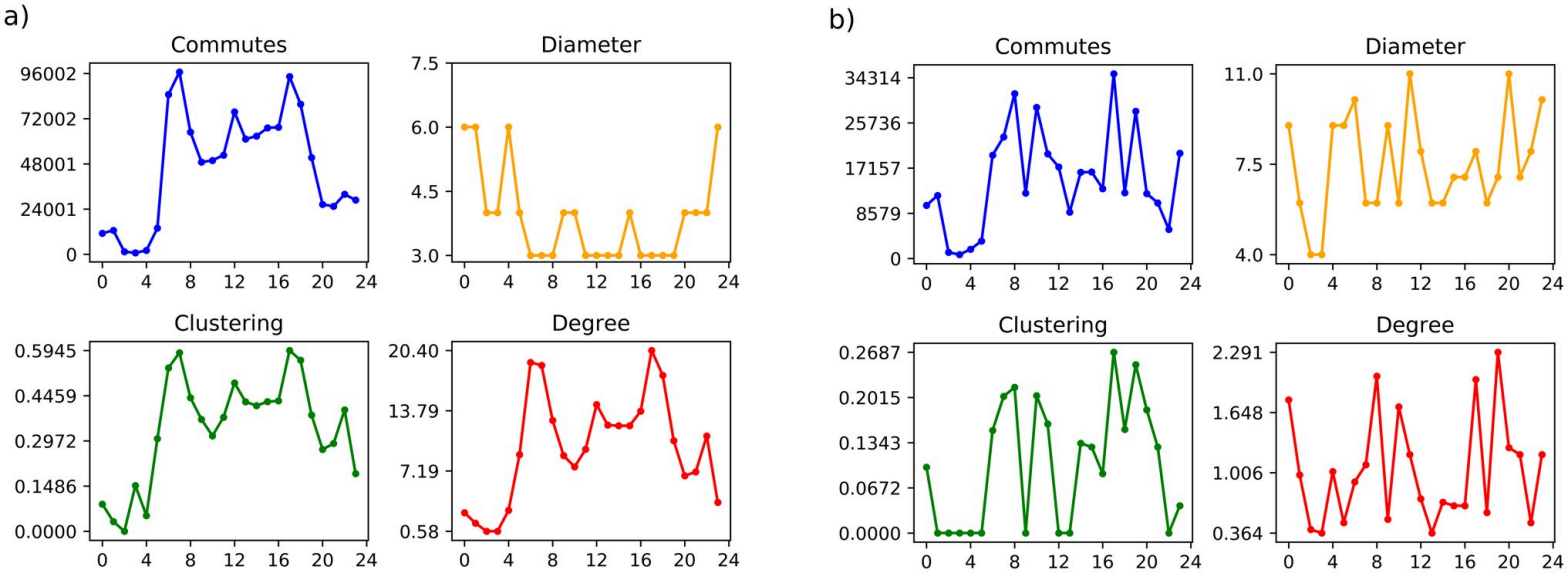
Time series of topological indexes for the set of networks with (A) *η* = 1 and (B) *η* = *ηc*.

Fig 4(A) represents the behavior of *η* equals 1, in which the agglomeration, degree, and travel present the same behavior in the temporal evolution. Fig 4(B) represents the behavior of *ηc* equals 560, in which even with the high reduction of connections, the high flow travel times were preserved. The *ηc* is chosen accordingly to the method presented in the previous section, where it is defined from the diameter value. This method is applied to each time window.

The time series for the average topological degree and the average clustering coefficient are quite similar to the commutes’ time series. The time series for the diameter is inversely related to the previous ones. With Â *etac*, structured the network from the largest diameter of Â *eta* = 1, so reduced the number of connections between TZs, but preserved the most important edges, which already expected it [16, 33].

The average topological degree (〈*k*〉) stays less than one at 2 am, and 3 am. At 6 am and 7 am and 0, 5 and 6 pm, 〈*k*〉 is greater than 14, the average clustering coefficient (<*c*>) is greater than 0.45. For all these periods, the diameter (D) is 3, the lowest value for the 24 networks (Fig 4A).

Commutings from residence to work begin to grow at 6 am, but the peak hours occur between 7 am and 8 am. The reverse movement’s peak time range, from work to residence, is between 5 pm and 6 pm. These commutes are well defined and show that most people leave the house to work in the morning and return home at the end of the day.

Commutings from home to school generate three peaks in this sequence in relation to the number of commutes: the first between 6.30 am and 7 am; the second between noon and 1 pm; and the third at 6 pm. The reverse movement, from school to residence, also generates three peak hours, in this sequence in relation to the number of commutes: the first at noon; the second close to 5:30 pm; and the third one near 10 pm. It is worth noting that it is common in Brazil to have higher education courses at night to meet people who need to work during business hours. Unlike work commutes, study commutes are distributed throughout the day since most students remain only one period of the day at school. The peak of most significant amplitude on the way at 6:30 am, and the one at the back, at noon, show that the morning period concentrates the largest number of students.

Fig 4(B) brings the time series of topological indexes for the set of networks with *η* = *ηc*, for each time window. *ηc* varies from 9 to 560, and its average value is 300. From a general point of view, changes in the time series of topological indexes are more significant under the critical connection threshold than under the less restricted one (*η* = 1). However, the variation/amplitude in this case, *η* = *ηc*, for 〈*k*〉 and <*c*> are lower (2.3 and 0.27, respectively) and for *D* (11) is greater than for that case, *η* = 1.

**Fig 5:**
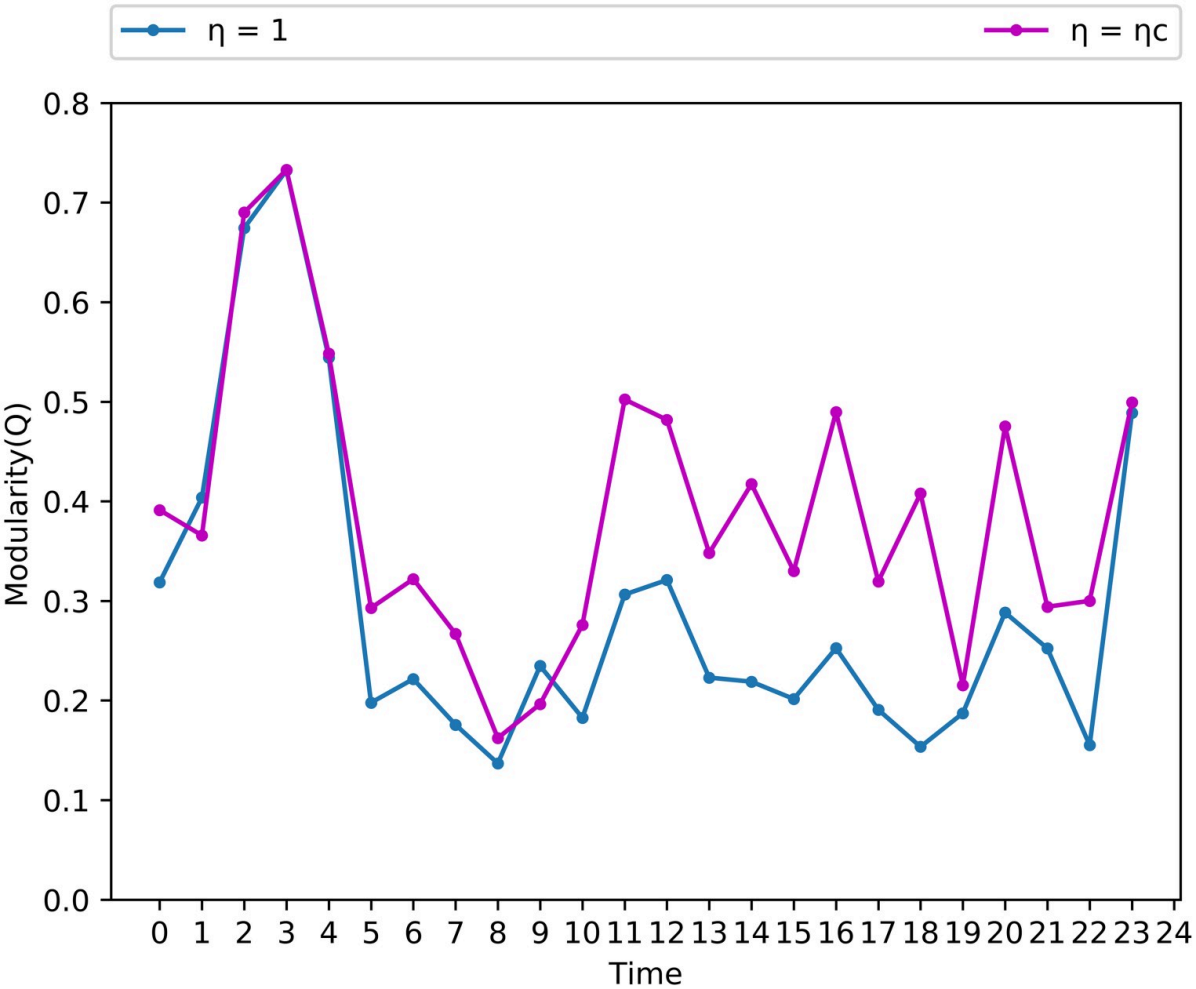
Time series of modularity *η* = 1 and *η* = *ηc*.

For *η* = 1, the community structure is clear between 2 am and 4 am and at 11 pm, only (Fig 5). For *η* = *ηc*, Fig 5 shows the time series for the modularity index. It is important to highlight that index is greater than 0.3 for almost all time windows.

**Fig 6:**
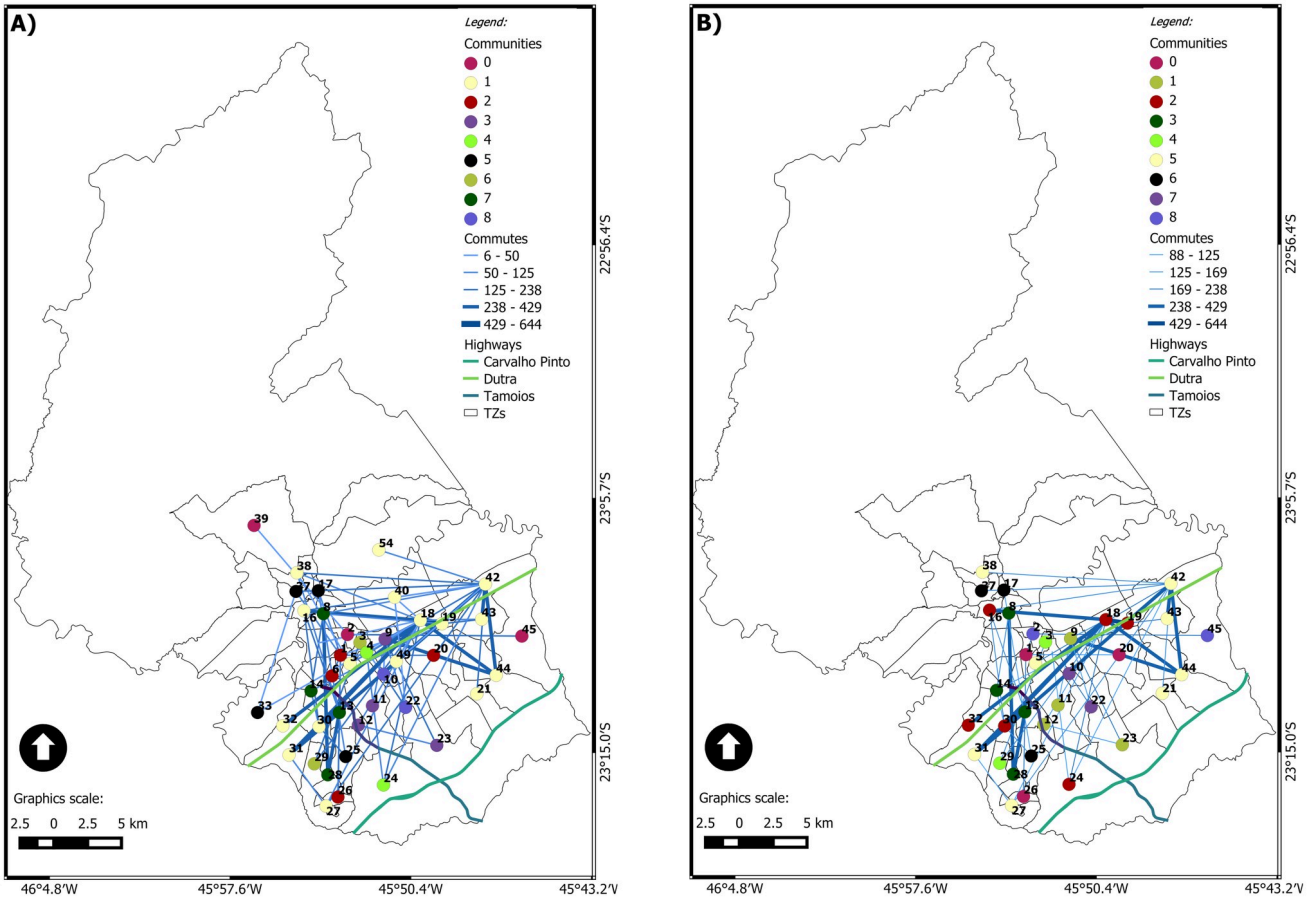
(Geo)graph for the dynamic network at 0 am. (A) *η* = 1 and (B) *ηc* = 88.40.

**Fig 7:**
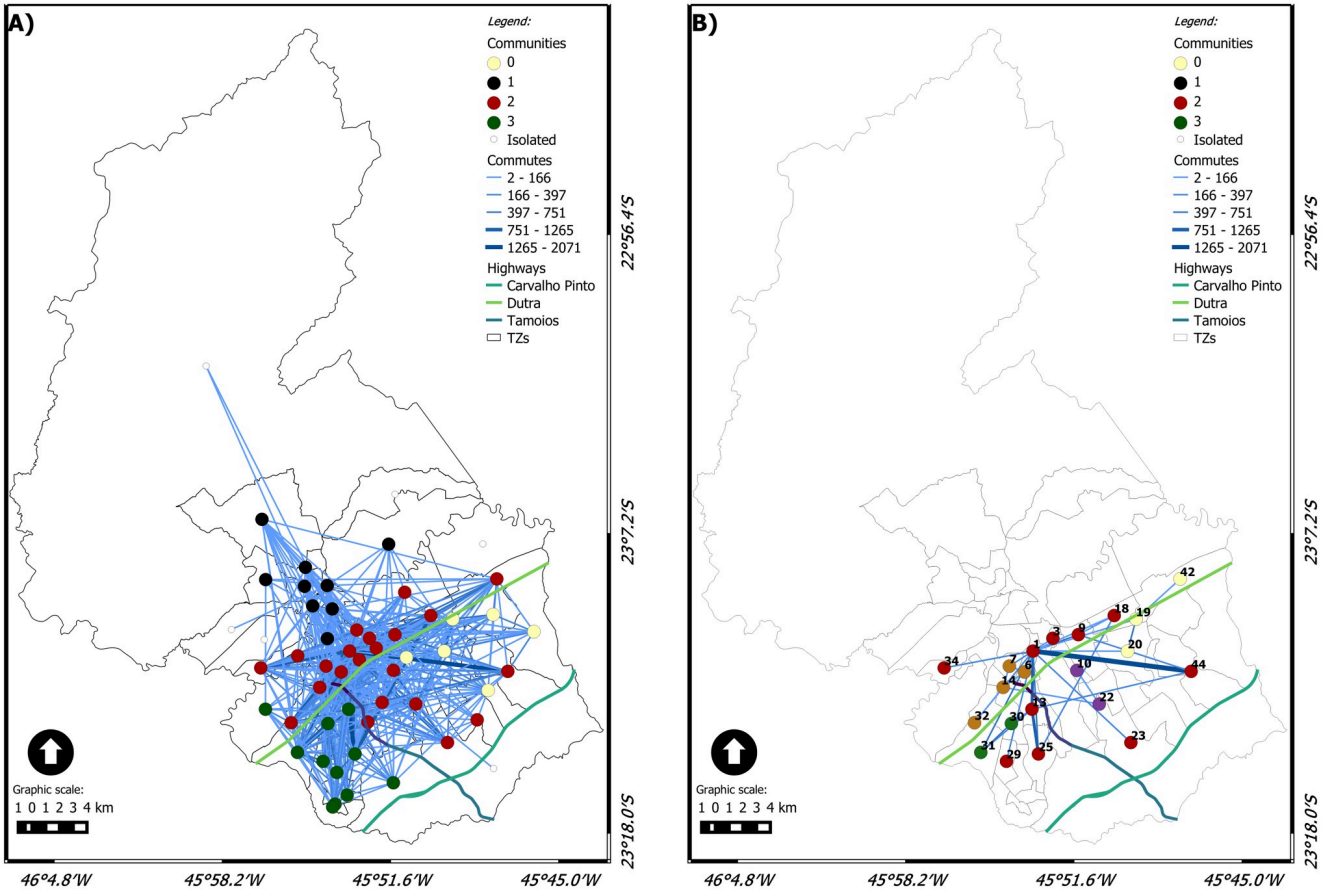
(Geo)graph for the dynamic network at 7 am. (A) *η* = 1 and (B) *ηc* = 510.

**Fig 8:**
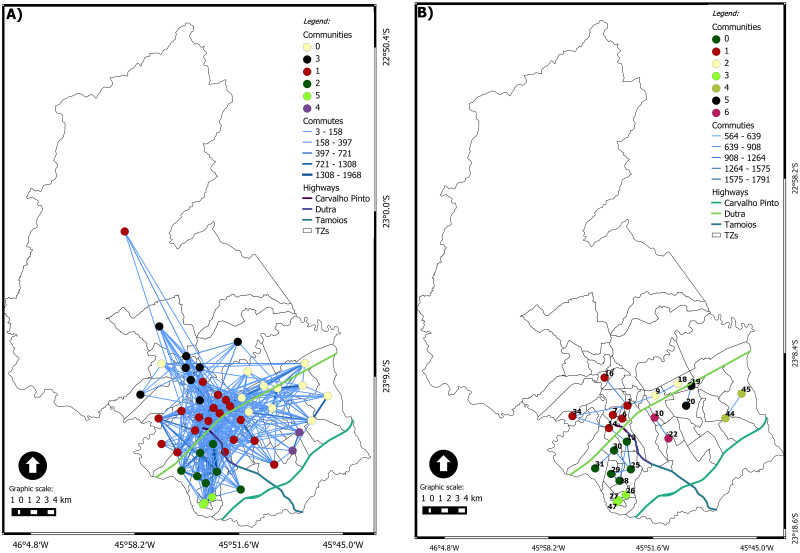
(Geo)graph for the dynamic network at 12 am. (A) *η* = 1 and (B) *ηc* = 564.

**Fig 9:**
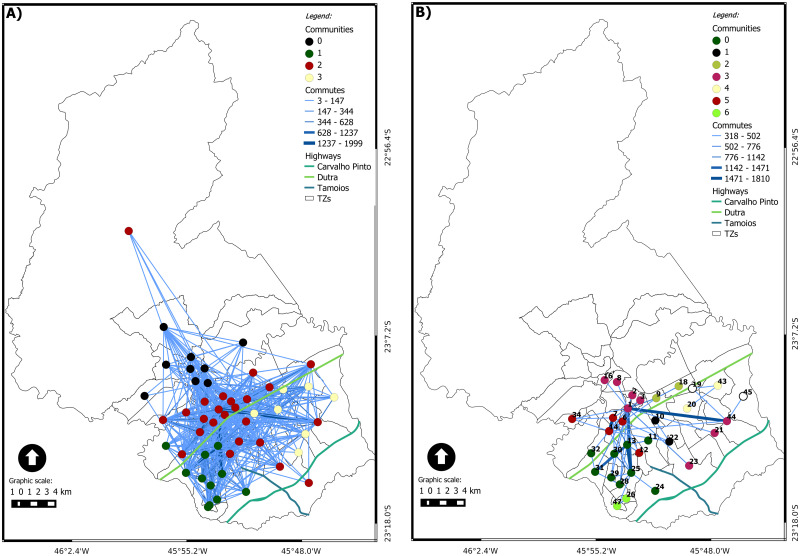
(Geo)graph for the dynamic network at 5 pm. (A) *η* = 1 an (B) *ηc* = 318.

**Fig 10:**
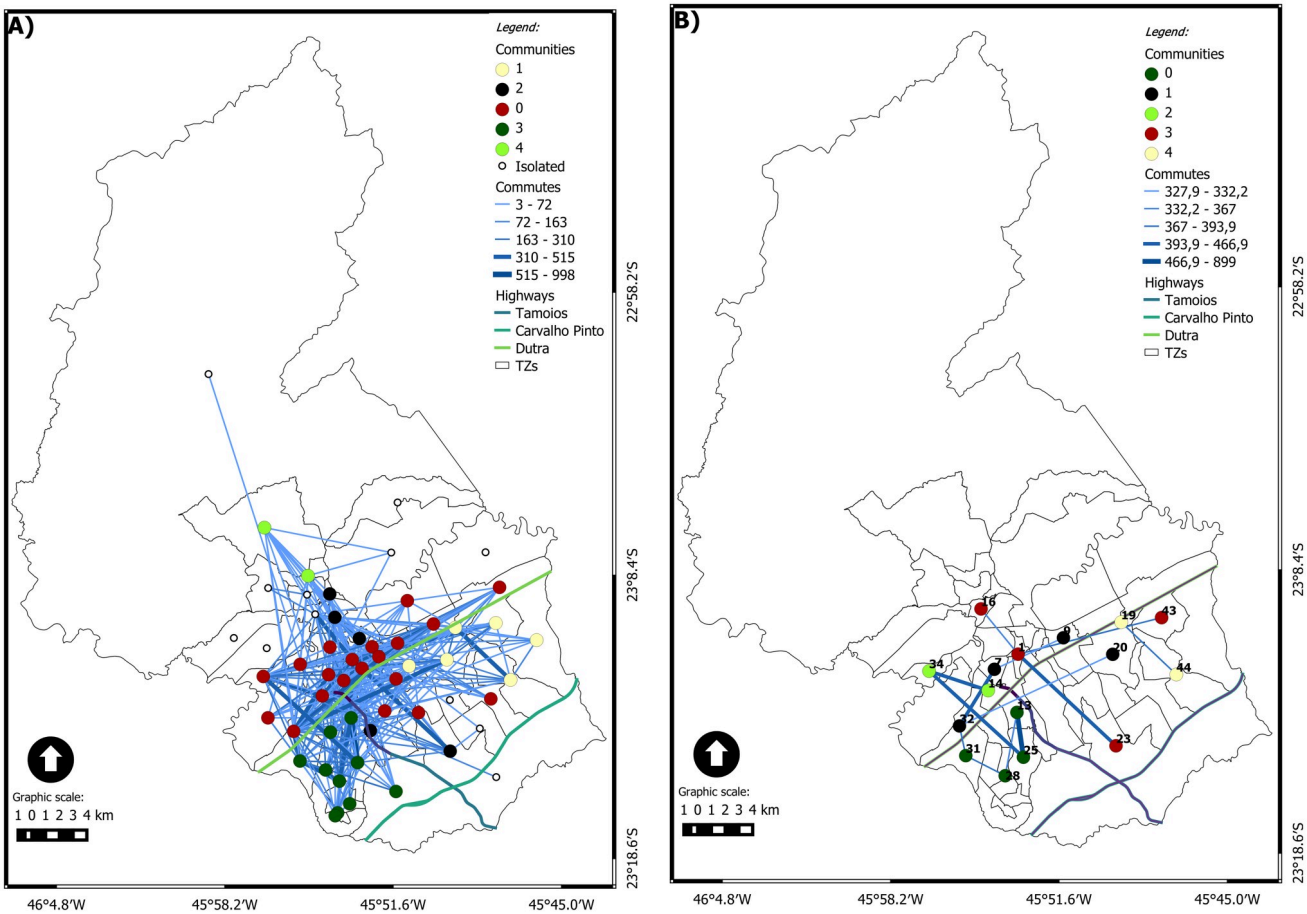
(Geo)graph for the dynamic network at 10 pm. (A) *η* = 1 and (B) *ηc* = 327.

Figs 6, 7, 8, 9 and 10 show the dynamic network, following the (geo)graphs approach, with *η* = 1 in left side and *ηc* in the right side, for different time windows: 0*am*, 7*am*, 12*am*, 5*pm* and 10*pm*, respectively. Our most important findings are:

- 0 am (Fig 6): the largest community has 16 nodes for *η* = 1 and 8 for *ηc* = 88.40. In both cases, it is spatially spread on the city. The critical connection threshold *ηc* = 88.40 is a very low value compared to the average connection threshold for the dynamic case (314). The midnight commutes are composed, almost in its entirety, of commuters who leave work and are returning home. The zones with the highest number of commutes at this time are, respectively: 18 (named Vila Tesouro) where the General Motors plant is located and 42 (named Eugênio de Melo) where the Ericson plant is located. The total commutes from zone 18 are almost double the total commutes from zone 42. The highest edge on the network is from TZ18 to TZ19 with 644 commutes.
  For the first hour of the day, the community with the highest number of vertices is community 1, composed of 16 vertices with industrial, central and residential areas, as Fig 6(A) shows. Fig 6(B) presents nine communities for *ηc* = 88.40, where two zones have grade 11, with high number of travel between industrial regions. Both zones have five connections in common.
- 7 am (Fig 7): There are *four* communities for *η* = 1 and *five* communities for *ηc* = 510. The largest community for each case brings nodes in the central area but nodes away from that area. The nodes from the other communities are spatially close to each other. The critical connection threshold *ηc* = 510 is around the average connection threshold for the dynamic case (314). The main reason for commuting at 7 am is WORK (it represents 58% of the total commuting during that time), followed by STUDY (15% of the total).TZ 1 is the zone that attracts the most trips due to WORK. The travel attraction of TZ 1 comes from several macrozones. The Macrozona South also attracts a significant number of trips for work reasons. However, it has an attraction concentrated in neighboring or nearby areas.
- 12 am (Fig 8): the network has almost the same behavior as the previous schedule considering *η* = 1, highlighting only that the southern region is now divided into two communities. Community 0 represents the predominant community in the eastern region. Community 1 is predominant in the central region, consisting of 18 vertices, with only five located in the southeast and 1 in the extreme north. The southern region has two communities, 2 and 5; community 2 is a small, simply connected set, consisting of 3 vertices, 5 has seven simply connected vertices. Community 3 is predominant in the northern region, with only one vertex in the central region. Community 4 is a simply connected peer from the eastern region. When we consider *ηc* = 564, it is possible to observe that the city’s highways can be considered a community divider, presenting a geographical influence. Two communities stand out, both with six vertices. Community 0 has a set of related vertices in the southern region of the city. The maximum distance between two pairs of vertices is 4.3 km.
- 5 pm (Fig 9): this time window has the most significant number of trips. The community detection is similar to what was presented at 7 am, considering *η* = 1. Community 0, with seven vertices, is predominant in the eastern region. Community 1, with nine vertices, is predominant in the northern region. Community 2, with 24 vertices, runs from east to west, growing from the north direction of the highway throughout the city. Considering *ηc* = 318, the community of the southern region increases to 9 vertices, adding a vertex from the south region itself, one from the west region, and one from the southeast region. The longest distance between the vertices is 11.6 km from community 3. The critical connection threshold *ηc* = 318 is around the average connection threshold for the dynamic case 314.5.
- 10 pm (Fig 10): five communities are detected with *η* = 1. Compared with the last time window, a new community appears in the southeastern region, with zones belonging to the northern and central regions. The community 0 has 20 vertices, has 16 vertices on the same side of the highway (north), the vertices follow the entire highway, from east to west. The other vertices follow the highway in a southeast direction. Community 1 has six vertices, and it is concentrated in the eastern region below highway Dutra. Community 2 has two disconnected sets of connected regions. Community 3 is composed of ten vertices in the south, all connected. Community 4 has a connected pair in the extreme north. Although the community in the southern region suffers a decrease in the number of vertices, considering *ηc* = 327, it remains connected, with two vertices communicating with external communities. The zones belonging to community 1 are in the north, east and southeast regions. There are two most prominent communities: one community related to the geographical context with areas in the southern region; another behaving like *hub* being the center zone with long edges and high flow. There are two private universities, Unip and Univap, with evening courses at TZ 34. The community formed in this western region has two nodes, highlighting the number of trips between zones 34 and 14, with the city’s highest per capita income.

## 4 Conclusions

This work’s goal was to present static and dynamic networks for urban mobility networks using the OD database as input. The initial threshold, considering all trips, and critical threshold were explored to create connections and establish a network topology. Thus, a study of the network’s topological measures and community structures was carried out. Their evolution was presented over time, revealing the patterns and persistence of the space-time dynamics. This approach was applied in the municipality of São José dos Campos, located in the state of São Paulo, southeastern Brazil.

In the measures of centrality and connectivity at the initial threshold of the static network, a highly connected network (with 〈*k*〉 equals 32.7) was observed, that is, there are many trips between all other areas of the city. TZ 1 (called downtown) stands out with a high degree of connection, with 23% of total trips. When applying the critical threshold, only 11 zones remain connected with a 〈*k*〉 of 0.36 and a total of 133,448.62 trips. TZ 1 stands out again with approximately 22% of all trips, connects to 4 zones, and starts to behave like a hub in the network. Thus, the TZ 1, regardless of the threshold, proved to be a zone of great represent activeness both for its number of trips and for the connection between zones.

We have selected and scrutinized five networks (0am, 7am, 12am, 5pm, and 10pm) for the dynamic network, representing the commuting peaks in the interval. For these networks, we have depicted their main characteristics. The time window starting at 0am has a small number of trips and differs from the other intervals. This network is more connected and shows trips among various zones. At 7am, the network with an initial threshold shows four well-defined community structures similar to the one presented in the static network. There was an addition of two communities in the 12am network for the initial threshold network. In the southern region, the community was divided into two. Each new community has pairs of zones with many trips, suggesting that the displacement was not sparse for this region and time. About the 5pm the network, the results were similar to the network at 7am. Finally, at 10pm, the network depicted topological values and community structures related to places such as shopping centers, universities, and the industrial area. In the case of the critical threshold, network connectivity decreased at all times. As in the static network, TZ1 remained strongly connected regardless of the time, albeit TZ1 was not the major hub in all scenarios.

For community structures, the city’s southern region had little variation throughout the day, showing that it is an area that suffers little influence from other regions of the city. Another important structure was the extreme north’s apex, which is highly dependent on the central region of the city and not on its own geographical region. This outcome might suggest a different perspective of the geographical context and its relation to the mobility network. From the modular structure, it can be highlighted that the modularity values for the critical threshold are higher than those of the initial threshold. Another important consideration concerns the Presidente Dutra highway, which is configured as a strong physical barrier for urban mobility, especially for those who commute between traffic zones that compose a community. This feature was observed in networks with distinct thresholds and time.

The communities’ analysis also showed the existence of four well-defined communities in the Central, South, East, and North regions. The predominant community is in the central region. The southern region presented a well-defined structure at different times, but with stronger evidence at 7am and 5pm. At noon, the community gains new TZs on both sides of the Presidente Dutra Highway, which used to be in the central region, indicating that commutes prefer nearby locations. For times of low commute flows, specific characteristics are essential for the definition of the community. At 0 am, the community is defined by the influence of industries with commutes work-home.

Results have shown that analyzing the geographical data aggregated in the space-time dynamics allowed interpretations with a different perspective of urban mobility. This new perspective can assist in the decision making of public managers for, for example, urban planning and communication networks and assist in studies of the spread of epidemics and extreme events such as disasters.

As a direction for future work, this approach can be directly applied to other cities or be extended with further data. A current example can be mobility studies aggregated with the study of the spread of epidemics, such as SARS-CoV-2, for actions to prevent contamination.
